# Progress in Medicine

**Published:** 1895-05-04

**Authors:** 


### PROGRESS IN MEDICINE.

### RENAL MEDICINE.

**Suppression of Urine.**—The complete suppression of urine without symptoms of uræmia has been often the subject of speculation. Bampton of Ilkley[1] mentions an instance where suppression lasted ten days without convulsions, and discusses three theories to account for it: (1) "choked filter" from accumulation of excrementitious bodies; (2) renal paralysis from an alkaloid poison; and (3) spasm of the renal artery from irritation by urinary poisons. He thinks that in his case potassium salts were the active agent. Jessop, in connection with this instance speaks of a man—now alive and well—who twice suffered from complete suppression for ten days, with sudden recovery. Many of these patients appear perfectly well until near the end, when some twitching or convulsions come on. Duckworth, in a clinical lecture, refers to two cases which lasted respectively twenty-one and twenty-five days. Besides the commonly recognised causes, such as shock, enteritis, poisoning, and obstruction by a calculus when there is only one active kidney, Duckworth notices[2] that occasionally in chronic nephritis the kidney ceases to act from some unknown reason. This happened in a case under his care with advanced parenchymatous and possibly some interstitial disease. The hysterical cases, of which Hensley records an instance,[3] have been regarded as presenting a vicarious elimination of urea through the intestinal tract, but the whole subject is one of much difficulty. Suppression for eight days, followed by a sudden copious diuresis in a case of malignant uterine disease, reported by J. J. Stack, would probably depend on obstruction.[4]

**Uræmia.**—Hughes and Carter report the results of their investigations on uræmia, but hardly seem to recognise the great variety of diseases included under that name. They regard it as produced by a poison, probably albuminous in nature, affected by heat and dialysable with difficulty.[5] This substance is not urea, and has the property of producing nephritis and fatty degeneration of the retina. It is possible sometimes to have uræmia without previously existing lesions in the kidneys. In such instances they believe that there is a sudden excessive production of the poison. Uræmia, again, may be accompanied at times with a rise of temperature, especially when parenchymatous disease of the kidneys is present. Injection of urine or its ordinary constituents does not produce uræmia, though ligature of the renal arteries does. Transitory blindness, occurring like transitory hemiplegia in uræmia, is considered by Rothman to depend on œdema of the sheath of the optic nerve rather than on that of higher centres. The pupil reaction may be retained or lost, but the prognosis is more favourable when it remains intact. Chronic nephritis without uræmia may produce the same results.

Pericarditis, too, may develop in uræmia, either septic or otherwise in nature. Beco[7] refers some of these attacks to the bacillus coli, others to the presence of toxines, and others to non-microbial causes. Pressure on the common carotids, repeated at intervals, is recommended by V. E. H. Lindsay[8] as a means of checking uræmic convulsions quite as efficient as the administration of chloroform. Two cases of detachment of the retina in the course of granular kidney were detailed at the Clinical Society by Dr. West.[9] This appears to be a very rare complication. The prognosis in all these cases of albuminuric retinitis is, he considers, extremely grave, life being rarely prolonged more than a few months. Hale White had seen retinitis, where there was no loss of vision, clear up, so that the eye appeared later on to be normal. There would appear to be cases of retinitis in children which are exactly similar to the albuminuric type though no disease of the kidneys exists.

**Cardiac Hypertrophy in Bright's Disease.**—De Dominicis, to investigate the production of cardiac hypertrophy in Bright's disease, tied the renal artery on one side in twenty animals.[10] A month later no alteration in the heart could be detected, but epithelial and interstitial changes were found in both kidneys, though no albumen or casts were present in the urine. Hence, he argues, if cardiac hypertrophy and renal disease occur together they are both probably the results of a chemical poison in the blood. The hypertrophy is not the effect of the renal changes. According to Enriquez and Hallion, injections of diphtheritic toxine in a monkey produced many months after, not only various nerve degenerations, but also granular kidney and cardiac hypertrophy.[11] There were no arterial lesions accompanying the kidney changes, nor was there any sclerosis of cardiac tissues.

Was then the hypertrophy merely functional and the result of the kidney changes, or can we suppose with Dominicis that the toxines could in any way cause the heart change?

**Renal Dropsy.**—In discussing the cause of renal dropsy, Senator[12] singles out four types of disease as those in which it most frequently occurs, viz., nephritis *a frigore*, that in scarlatina, that in malaria, and that in pregnancy. In these the glomeruli are first affected, in others the tubules. Hence it appears that diseases of the former are more likely to lead to inflammatory changes in the vessels than those of the tubules are, and so to produce those changes in the vessels which permit of transudation. Chronic parenchymatous nephritis is no exception, for the glomeruli are involved as well as the parenchyma, and, in short, Senator holds that renal dropsy is a result of certain lesions in the vessels of the kidneys. In place of Southey's tubes and other methods of treatment for anasarca, Leichenstern[13] now makes a single incision of three centimetres in length below the external malleolus, or on the dorsum of the foot through the whole thickness of the skin. The flow of fluid is four times as abundant in this method as when other plans are employed. The patient is placed in an arm-chair, if able to sit up, with his legs previously asepticised and the feet supported on a stool in a footbath. The edges of the wounds are coated with vaseline, and a perchloride compress wrapped round the feet, which are enveloped in a rubber sheet, draining the fluid into the bath. Once a day the wounds are washed out, and when it is desired to close them a tight dressing is applied. No excoriations or rashes appear under this treatment.

**Palpation of the Kidney.**—The question of the palpation of the kidneys in normal conditions has of late advanced towards settlement. When the two hands are employed and the patient takes a quick, deep breath, the right kidney may often be felt coming down between them, sometimes for half its length, sometimes presenting its tip, and sometimes causing only an increased resistance from above. This is true less frequently of the left kidney, but in all cases there must be some degree of relaxation of the abdominal walls and only a scanty amount of fat. A. M. Stalker[14] estimates that in half the multiparous women in a ward the right kidney can be definitely palpated, and in a sixth of these the left may be clearly felt also. In nulliparæ and in children success is rarer, and in males only a few extremely wasted patients have kidneys which can be accurately palpated. Skene Keith, however, speaks of palpation as possible in all cases, except those where there is an abnormal amount of fat. Stalker would explain this as meaning that an increased resistance in inspiration can be felt, which does not include the perception of the outline of the organ, though even this pressure feeling has some degree of diagnostic value. Percussion would seem to be of little practical value.

**Ether-anæsthesia; its Effect on the Kidneys.**—Additional evidence of the harmlessness of ether-anæsthesia to the kidneys is given by Barensfeld. Out of 150 operation-patients only four showed albumen after the operation. Two of these may be deducted as having albumen beforehand, but the amount was not increased when tested twenty-four hours after the operation, and the two in which it first appeared after anæsthesia were severe abdominal cases.[15] Still, we must place against these facts the cloudy swelling found in the kidneys during the experiments of G. B. Wood and others, especially after repeated or very prolonged administration of ether.[16] No elimination apparently takes place by the kidneys, and though the ether exists in a free state in the blood it passes off probably through the lungs alone. Pilocarpine, too, when applied locally by friction on the trunk, is said to stimulate the kidney by a nerve reflex, though it is not absorbed or eliminated in the urine.[17] Mollière claims a high value for it in nephritis when thus applied, as it produces copious sweating and moderate diuresis without disordering the stomach. He rubs in an ointment containing from four- to eight-fifths of a grain of pilocarpine, and covers the skin with cotton wool and waxed linen.

**Urine Analysis.**—Indican: The contributions to the question how far its presence is a sign of tuberculosis in children include papers by Gehlig and Cima, whose results show that the indican reaction depends more on some irregularity in feeding than on tuberculosis, and is important only as showing the amount "of decomposition of albuminoid substances in the intestines."[18] Cystin is estimated by very complicated methods, of which Borisson's is, perhaps, the most recent. It consists[19] in precipitation by HCL, zinc, mercuric chloride, and sodium acetate, followed by purification and estimation of the cystin by the amount of sulphur present. A case of cystinuria is reported by A. J. Hale.[20] Cystic calculi had been passed at intervals for ten years, and the patient complained of much lumbar pain, with attacks of colic. Great relief and disappearance of cystin crystals from the urine was obtained while he was kept under the influence of alkalies in large doses, so as to render the urine alkaline. It seems quite possible that the estimation of cystin and other sulphur compounds may be found clinically useful in the future, not only as affording a measure of the metabolism of the proteids of the body, but also as a measure of the activity of the liver and other organs. These compounds are either sulphates, or less oxidised bodies, such as sulphocyanates and cystin; and intestinal decomposition, or such poisons as phosphorus and pyro-gallic acid, may markedly reduce the sulphates and increase the other compounds by inhibition of the liver.[21]

**Hæmato-porphyrin** in urine has become of importance, partly from its somewhat frequent occurrence in persons taking sulphonal. Priestley gives an exhaustive account of the whole subject. Its solutions possess a peculiar spectrum, acid ones having two bands between D and E, and alkaline ones four between C and F. It has been found in many diseases, and in small amount as a normal constituent in health by Garrod and others. Occasionally in typhoid, gout, rheumatism and liver diseases, the urine is dark-coloured from its presence, and a similar state may appear suddenly in other persons, especially women, with a group of obscure nervous symptoms. Hæmato-porphyrin may persist for a long while without affecting the general health, but in other cases, especially the neuropathic ones, rapid and fatal exhaustion may follow. Priestley shows that the connection between it and sulphonal is doubtful. Often the sleeplessness for which sulphonal is taken is an early symptom of the states in which this substance appears. Still sulphonal must be given with care in such patients, as about forty instances with twenty deaths have occurred in which its appearance followed the use of the drug.[23] Vomiting, constipation, diminution of urine and abdominal pain may mark the commencement of an attack, with sometimes motor paralysis. There is some reason, according to Oswald, to believe that degeneration of the adrenal bodies is present in these cases—kidney disease may be a consequence of the attack, but these organs are often quite normal.

**Peptonuria,** according to Senator,[24] who agrees with Stadelman and others, is really an albumosuria, and occurs very frequently in pneumonia, purulent meningitis, and empyæma, sometimes in acute rheumatism, but not in leucocythæmia. It is a valuable aid to diagnosis, and may be best determined by Salkowski's method of precipitation by phospho-molybdic acid and biuret. True peptone is only formed by the decomposition of albuminous urine. Alkaloidal bodies of varying toxicity have been found in the urine by Albu in phthisis, tetany, Grave's disease, and other affections, but their classification is still uncertain.[25]

**Albuminuria.**—Semmola[26] still argues for the view that albuminuria is based on a morbid diffusibility of the sero-albumen, produced by a molecular alteration in the blood, at least in the majority of cases. When, in addition, a nephritis exists, it is a result of the existing diathetic condition, such as gout, an acute infectious disease, or other poisonous agent. A small amount of albumen from nephritis where no such dyscrasia exists may indeed occur, but the larger amounts in true Bright's disease depend on the changes in the blood, and are a useful elimination of a substance which has become heterogeneous and toxic. He relies very much on the absence of any fixed relation between the degree of albuminuria and the amount of the changes found in the kidneys—which is undoubtedly a strong point—and claims that continuous injections of egg-albumen produce progressive albuminuria, secondary nephritis, œdema, diminution of urine, and albuminuric retinitis. The cause of Bright's disease and the preceding dyscrasia of the blood is, however, as yet quite unexplained. He meets with more general assent in urging that our treatment should be directed rather to improving the general health than to reducing the excretion of albumen. Everyone admits that diseased kidneys will perform a sufficient amount of work for surprisingly long periods if the metabolism of the body generally is efficient.

[1] Lancet, Dec. 15, 1894. [2] Clinic. J., Aug. 15, 1894. [3] Clinic. J., Oct. 10, 1894. [4] Lancet, Jan. 12, 1895. [5] Am. J. Med. Sc , Sept., 1894. [6] Pract., Dec., 1894. [7] B. M. J., Nov. 10, 1894. [8] Med. Week, Sept. 14, 1894. [9] Lancet, Feb. 2, 1895. [10] Pract., Jan. 10, 1894. [11] Med. Week, Dec. 14, 1894. [12] Med. Week, Feb. 22, 1895. [13] Med. Week, Feb. 8, 1895. [14] Ed. Med. J., Sept., 1894. [15] N. Y. Med, J., Jan. 5, 1895. [16] Am. M. S. Bulletin, Oct. 15, 1894. [17] N. Y. Med. J., Feb. 5, 1895. [18] Am. J. Med. Sc., Jan. 5, 1895. [19] N. Y. Med. J., Feb. 9, 95. [20] Med. Quarterly, Oct., 1894. [21] Arch. de Physiol., Jan., 1895. [22] Med. Chron., Aug.-Sept., 1894. [23] Glasgow Med. J., Jan., 1895. [24] Med. Week, Mar. 22, 1894. [25] B. M. J., Jan. 19, 1895. [26] B. M. J., Feb. 23, 1895.

